# Cell Proliferation Is Strongly Associated with the Treatment Conditions of an ER Stress Inducer New Anti-Melanoma Drug in Melanoma Cell Lines

**DOI:** 10.3390/biomedicines9020096

**Published:** 2021-01-20

**Authors:** István Szász, Viktória Koroknai, Vikas Patel, Tibor Hajdú, Tímea Kiss, Róza Ádány, Margit Balázs

**Affiliations:** 1 MTA-DE Public Health Research Group, University of Debrecen, 4032 Debrecen, Hungary; szasz.istvan@med.unideb.hu (I.S.); koroknai.viktoria@med.unideb.hu (V.K.); adany.roza@med.unideb.hu (R.Á.); 2 Doctoral School of Health Sciences, University of Debrecen, 4032 Debrecen, Hungary; vikas.patel@med.unideb.hu; 3 Department of Anatomy, Histology and Embryology, Faculty of Medicine, University of Debrecen, 4032 Debrecen, Hungary; hajdu.tibor@med.unideb.hu; 4 Department of Public Health and Epidemiology, Faculty of Medicine, University of Debrecen, 4032 Debrecen, Hungary; kiss.timea@med.unideb.hu

**Keywords:** HA15 anti-melanoma drug, ER stress, apoptosis, autophagy, resistant cell lines, RNA-Seq

## Abstract

HA15 is a new anti-melanoma drug that triggers endoplasmic reticulum (ER) stress and causes deleterious effects on melanoma cell viability due to autophagy and apoptosis, regardless of driver mutations or drug resistance. In this study, we investigated the effect of HA15 on the viability/proliferation of *BRAFV600E*-mutant melanoma cells using different culture conditions. In contrast to the published data, we did not detect significant melanoma cell death under normal culture conditions using HA15 treatment. Indeed, only cells that were cultured under long-term starvation conditions were sensitive to the drug. Quantitative measurements of ER stress and autophagy markers showed that the compound HA15 does not trigger stress alone but synergistically enhances ER stress under starvation conditions. Importantly, we observed that the viability of normal melanocytes decreased significantly with treatment, even at low HA15 concentrations. Finally yet importantly, we were able to generate HA15-resistant cell lines, which failed by Cerezo et al. In summary, HA15 only influences the viability of cells that are starved for several hours before and during treatment. However, this in vitro setting is far from the in vivo conditions. In addition, our data clearly show that melanoma cells can acquire HA15 resistance. Further studies are needed to prove that HA15 is an effective anti-cancer agent.

## 1. Introduction

Cutaneous melanoma is an aggressive malignancy with a high invasive capability, resulting in metastases in different organs. Although melanoma represents only 5% of cutaneous cancers, it is responsible for almost 75% of all skin cancer deaths [1]. It is well known that approximately 50% of metastatic melanomas harbor *BRAF* oncogene mutations, which lead to the uncontrolled activation of the mitogen-activated protein kinase (MAPK) signaling pathway [2]. Small-molecule BRAF and MEK inhibitors (vemurafenib, dabrafenib, and trametinib) have revolutionized treatment and have improved the progression-free survival of melanoma patients with advanced-stage and metastatic melanoma [3]. Furthermore, the number of effective drugs has expanded dramatically, including antibodies targeting immune checkpoint inhibitor molecules such as cytotoxic T-lymphocyte-associated antigen 4 (CTLA-4), programmed cell death (PD)-1, and PD-ligand1 (PD-L1) [4]. These advances in melanoma treatment have led to an increased median overall survival of patients with metastatic disease from ~9 months to over 2 years and, in some cases, have resulted in long-term remission [5]. Despite these changes in treatment options, more than 50% of patients still experience treatment failure due to acquired drug resistance to MAPK inhibitors and immune checkpoint blockade treatment [6].

Cerezo et al. recently synthesized and characterized a new molecule family, thiazole benzensulfonamides (TZBs), which have anti-cancer properties [7]. Based on their results, Cerezo et al. focused on one molecule of the family, named HA15, which was identified as the lead compound that induces elevated endoplasmic reticulum (ER) stress specifically in cancer cells without any adverse effects in normal cells [7]. Cerezo et al. showed that the drug induces the death of all melanoma cells independently of the cell mutational status. Similar observations were reported for freshly isolated melanoma cells, independent of whether patients were sensitive or resistant to BRAF inhibitors [7]. Cerezo et al. also identified the ER protein BiP/GRP78/HSPA5 as being a specific target of HA15, describing the fact that interaction between the compound and BiP (binding immunoglobulin protein) enhances ER stress and leads to melanoma cell death via the concomitant induction of autophagy and apoptotic mechanisms.

During cancer development, a significant amount of protein is required to support proliferation, migration, and differentiation in cancer cells [8]. The high rate of cancer cell proliferation results in a microenvironment with limited oxygen and nutrients due to inadequate vascularization. Therefore, cancer cells have to cope with hypoxia, pH variation, and nutrient deprivation, which leads to higher cellular stress than what occurs in normal cells [9,10,11]. The canonical unfolded protein response (UPR) pathway comprises three major transmembrane stress sensor proteins: PERK, IRE-1, and ATF6. The activation of these proteins is mediated by the master regulator chaperone BiP/GRP78/HSPA5. When a cell is challenged with ER stress, BiP dissociates from the three sensors and activates the UPR [12]. Accordingly, it is not surprising that, as a key molecule, BiP is overexpressed in many tumors, including melanoma, and is associated with higher tumor grades and reduced patient survival [13,14,15,16,17]. Hence, the UPR, and in particular BiP, seems to be a promising target in cancer treatment, with the goal of achieving a long-term response in patients with metastatic melanoma. It has been demonstrated that HA15 specifically targets the chaperone BiP/GRP78/HSPA5 and induces ER stress, leading to cancer cell death through the simultaneous induction of autophagy and apoptosis. Overall, HA15 exhibits strong anti-cancer effects in prostate, breast, colon, pancreas, glioma, cervical, and melanoma cells regardless of driver mutations or BRAF inhibitor resistance [7].

In this study, we sought to investigate the effect of HA15 on four *BRAFV600E*-mutant melanoma cell lines and their BRAF inhibitor-resistant counterparts established by our group [18]. In addition, we investigated the effect of HA15 on the most studied melanoma line A375 cells melanoma cell line as well as on normal melanocytes. To monitor HA15-induced ER stress and autophagy in cells, we examined which experimental conditions were the most effective to reduce cell proliferation after HA15 treatment. In addition, we successfully developed an HA15-resistant melanoma cell line after long-term drug treatment and highlighted some important limitations of using HA15 as a stress inducer.

## 2. Materials and Methods

### 2.1. Cell Culture

Melanoma cell lines (WM983A, WM983B, WM278, WM1617, and A375) were obtained from the Coriell Institute for Medical Research (Camden, NJ, USA). The cells were cultured in RPMI-1640 medium (Lonza Group Ltd., Basel, Switzerland) supplemented with 10% fetal bovine serum (Gibco by Life Technologies, Carlsbad, CA, USA), 2 mmol/L glutamine, and antibiotics at 37 °C. PLX4720 BRAF inhibitor-resistant cell lines were developed by our group [18] and cultured under the same conditions, but the medium was supplemented with 5 micromolar PLX4720 (Selleck Chemicals LLC, Houston, TX, USA). All the cell lines carried the *BRAFV600E* mutation and were wild-type for NRAS. The clinicopathological characteristics of the cell lines are summarized in Table 1. Melanocytes were isolated and cultured as described by Godwin et al. [3]. HA15 was purchased from Selleck Chemicals, Houston, TX, USA, and MedChemExpress LLC, Princeton, NJ, USA.

### 2.2. Cell Viability Assay

The WST-1 (2-(4-iodophenyl)-3-(4-nitrophenyl)-5-(2,4-disulfophenyl)-2H-tetrazolium) cell proliferation reagent (Sigma-Aldrich Inc., St. Louis, MO, USA) was applied according to the manufacturer’s guidelines. Briefly, melanoma cells were seeded in 96-well plates (0.5 × 10^4^ cells/well/100 µL medium) and treated with 10 µM of HA15 in triplicate for 48 h; DMSO was used as a control. In another experimental setup, we starved the cells without serum for 14 h before drug stimulation, after which the starvation medium was changed to normal medium containing HA15. In the third experimental setup, we starved the cells without serum for 14 h before drug stimulation, but we maintained the starvation conditions during HA15 treatment. In total, 10 µL of WST-1 was added directly to the culture in each well, and the cells were incubated for 3 h at 37 °C. Absorbance at 440 nm was measured using an Epoch™ Microplate Spectrophotometer (BioTek Instruments, Winooski, VT, USA). The reference absorbance was set at 700 nm. Cell viability was calculated by dividing the absorbance of the treated cells by that of the vehicle-treated (DMSO) control cells (considered 100%).

### 2.3. Development of an HA15-Resistant Cell Line

The *BRAFV600E*-mutant WM983B melanoma cell line was seeded at a low density in T/25 flasks until the cells reached 80% confluence. The medium was then switched to one containing 10 µM of HA15, and the concentration was increased to 30 µM of HA15 followed by culturing. For the surviving cells, a medium containing 30 µM of HA15 was added every 3 days until the cells reached 80% confluence (~10 weeks). The resistant cell line was designated WM983B^HA15RES^.

### 2.4. Drug Withdrawal Experiment

Melanoma cells (5 × 10^4^ cells/500 µL; WM983B^HA15RES^) were seeded in a 24-well plate (in triplicate) and cultured in RPMI 1640 supplemented with 30 µM of HA15 until attachment. Then, half of the cells was cultured in medium containing DMSO (the HA15 solvent), and the other half was cultured in HA15-containing medium for 72 h. WST-1 (50 µL/well) was added to the cell culture and incubated for 3 h at 37 °C. Absorbance was measured as previously described. The absorbance of the cells treated continuously with 30 µM of HA15 was considered 100%.

### 2.5. RNA Isolation and Real-Time Quantitative PCR

An RNeasy Mini Kit (Qiagen GmbH, Hilden, Germany) was used to isolate total RNA from the melanoma cell lines. The concentration and purity of the RNA were determined using a NanoDrop ND-1000 UV-Vis spectrophotometer (Thermo Fisher Scientific, Bioscience, Budapest, Hungary), and only samples with a ratio greater than 1.8 (260/280 nm) were included in the analysis. The reverse transcription of total RNA (600 ng) was performed using a High Capacity cDNA Archive Kit (Applied Biosystems, Foster City, CA, USA) according to the manufacturer’s instructions. qRT-PCR measurements were carried out in triplicate using the SYBR^®^ Premix Ex Taq™ master mix (Takara Holding Inc., Kyoto, Japan). The primer sequences of the candidate genes are listed in Supplementary Table S1. qRT-PCR was performed with a LightCycler 480 System (Roche Diagnostics GmbH, Mannheim, Germany). The PCR data were analyzed using the Livak method (2^−ΔΔCT^), and glyceraldehyde-3-phosphate dehydrogenase (Hs9999 9905_m1) served as the reference gene.

### 2.6. RNA Sequencing (RNA-Seq) and Data Analyses

The total RNA sample quality was determined using an Agilent BioAnalyzer with a Eukaryotic Total RNA Nano Kit according to the manufacturer’s protocol. Samples with an RNA integrity number (RIN) were accepted for library preparation. cDNA libraries for RNA-Seq analyses were prepared from 1 μg of total RNA using an Ultra II RNA Sample Prep kit (New England BioLabs Inc., Ipswich, MA, USA) according to the manufacturer’s protocol. Briefly, poly-A RNAs were captured by oligo-dT conjugated magnetic beads and the mRNAs were eluted and fragmented at 94 °C. First-strand cDNA was generated by random priming reverse transcription, and, after the second strand synthesis step, double-stranded cDNA was produced. After repairing the ends and the A-tailing and adapter ligation steps, adapter-ligated fragments were amplified by enrichment PCR; sequencing libraries were thus obtained. Sequencing runs were executed using an Illumina NextSeq500 instrument with single-end 75-cycle sequencing. Library preparations and sequencing were performed at the Genomic Medicine and Bioinformatics Core Facility of University of Debrecen.

The RNA-Seq raw data were deposited into the Sequence Read Archive database: (https://www.ncbi.nlm.nih.gov/geo/query/acc.cgi?acc=GSE164261) under accession number GSE164261.

The raw sequencing data (fastq) were aligned to human reference genome version GRCh38 using the HISAT2 algorithm, and BAM files were generated. Downstream analysis was performed using the StrandNGS software (www.strand-ngs.com). The BAM files imported into the software DESeq1 algorithm were used for normalization. To identify differentially expressed genes between conditions, we used the moderated t-test with the Benjamini–Hochberg false discovery rate for multiple testing correction. To gain mechanistic insight into the gene lists generated from the RNA-Seq data, a functional enrichment analysis was used to identify the biological pathways more enriched in a gene list than would be expected by chance. The ToppFun tool ToppGene suite (https://toppgene.cchmc.org/) was used to detect the functional enrichment of genes (which showed at least a 2-fold change difference between the treated and control groups) based on Gene Ontology (GO) pathways. The tool was used with default settings and a *p*-value cutoff of 0.05. For the visualization of the molecular functional gene networks, we used the ClueGo (v. 2.3.5) (8) tool kit of the Cytoscape (www.cytoscape.org) software (v. 3.5.1) with default settings and a p-value cutoff of 0.05. For a Gene Set Enrichment analysis (GSEA), we used the Molecular Signatures Database (MSigDB) hallmark gene sets, which summarize and represent specific well-defined biological states or processes and display a coherent expression (www.gsea-msigdb.org).

### 2.7. Flow Cytometry

WM983A cells were analyzed by flow cytometry using the Alexa Fluor 488 Annexin V/Dead Cell Apoptosis Kit (Invitrogen, Camarillo, CA, USA). The cells were treated with DMSO (control) and various concentrations of HA15 (10, 50, and 100 µM) for 48 h. Before drug treatment, we used starvation conditions (FBS was removed from the medium) for 14 h. The cells were harvested and washed with cold PBS. After centrifugation, the supernatant was discarded, and the cell pellets were resuspended in 1 × Annexin-binding buffer to a final concentration of 1 × 10^6^ cells/mL. After adding 1 μL of Alexa Fluor 488 Annexin V and 1 μL of 100 μg/mL propidium iodide (PI) working solution to each 100 μL cell suspension, the cells were incubated at room temperature for 15 min. After incubation, 400 μL of 1 × Annexin-binding buffer was added, and stained cells were detected by flow cytometry measuring fluorescence emission at 530 and 575 nM.

## 3. Results

### 3.1. Effect of HA15 Treatment on the Viability of Normal Melanocytes

**Figure 1:**
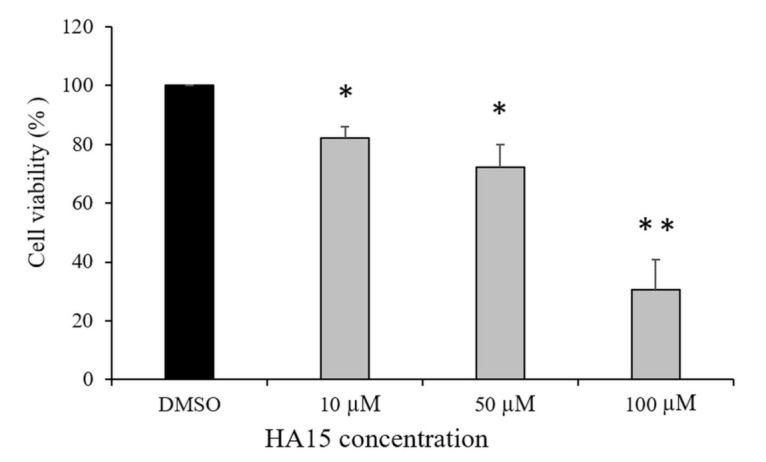
Viability of normal human melanocytes in response to increasing HA15 concentration under normal culture conditions after 48 h of treatment. DMSO is the solvent for HA15. The viability of cells was detected using the WST-1 assay. The data are presented as the mean ± SD of three independent experiments. Asterisks represent significant differences (* *p* ≤ 0.05; ** *p* ≤ 0.01).

To define the selective effect of HA15 on melanoma cell proliferation and viability, we first treated normal human melanocytes with the drug under normal conditions (cells cultured in the presence of 10% FBS) at different concentrations (Figure 1).

The viability of melanocytes decreased significantly (*p* ≤ 0.05) even after low-dose HA15 (10 µM) treatment, and increasing the drug concentration to 100 µM further decreased the cell viability under normal culture conditions (*p* ≤ 0.01).

**Figure 2:**
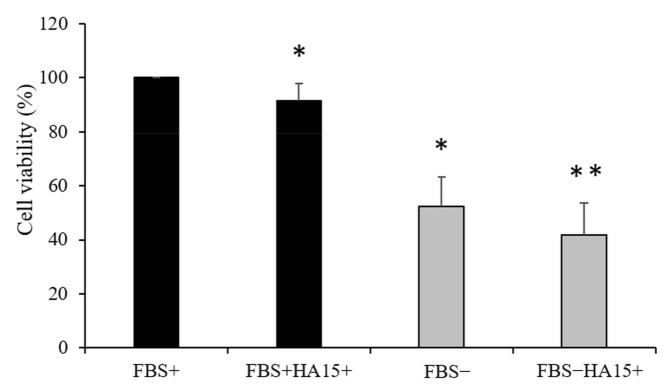
Viability of normal human melanocytes in response to 10 µM of HA15 treatment. First, cells were starved for 14 h and then cultured under normal culture conditions (black columns: FBS+) or cultured without FBS (grey columns: FBS−) or treated with 10 µM of HA15 (FBS+ HA15+ and FBS− HA15+) for 48 h. Data are presented as the mean ± SD of three independent experiments. Asterisks represent significant differences (* *p* ≤ 0.05; ** *p* ≤ 0.01).

In contrast to normal culture conditions, the viability of melanocytes that were starved before drug treatment (altogether 62 h) decreased below 60% without any drug, and adding the drug even at a low concentration (10 µM HA15) decreased the cell viability below 45% (Figure 2).

### 3.2. Effect of HA15 Treatment on the Viability of Melanoma Cells

We used two primary tumors (WM983A and WM278), two melanoma metastasis (WM983B and WM1617)-originating melanoma cell line pairs, as well as four BRAF inhibitor (BRAFi: PLX4720)-resistant cell lines (WM983A^BRAFiRES^, WM983B^BRAFiRES^, WM278^BRAFiRES^, WM1617^BRAFiRES^) to define the effect of HA15 on melanoma cell viability. Similar to the original publication, we also included the A375 melanoma cell line to compare our results to the published data [7].

**Figure 3:**
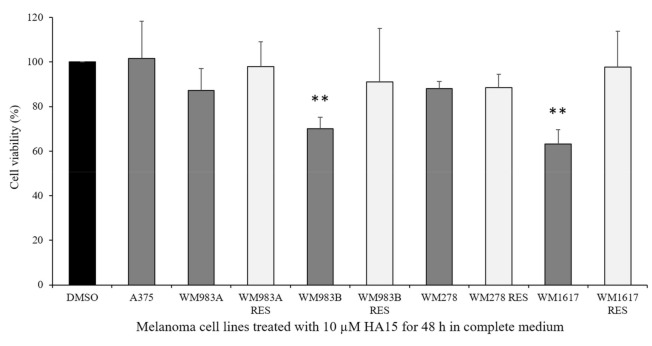
Cell viability of melanoma cell lines (A375, WM983A, WM983B, WM278, WM1617) and their BRAFi-resistant counterparts (WM983A^BRAFiRES^, WM983B^BRAFiRES^, WM278^BRAFiRES^, WM1617^BRAFiRES^). All the cell lines were treated with 10 µM of HA15 for 48 h in complete medium. Data represent the mean viability of three independent experiments (±SD). Significant differences are indicated by asterisks (** *p* ≤ 0.01).

We cultured all the melanoma cell lines under normal cell culture conditions and treated them with 10 µM of HA15. The viability of the cell lines was similar; however, two metastasis-originating cell lines (WM983B and WM1617) showed decreases below 70%. Interestingly, the viability of all BRAF inhibitor-resistant cell lines was close to that of the control, though the viability of the A375 cell line did not change at all (Figure 3).

**Figure 4:**
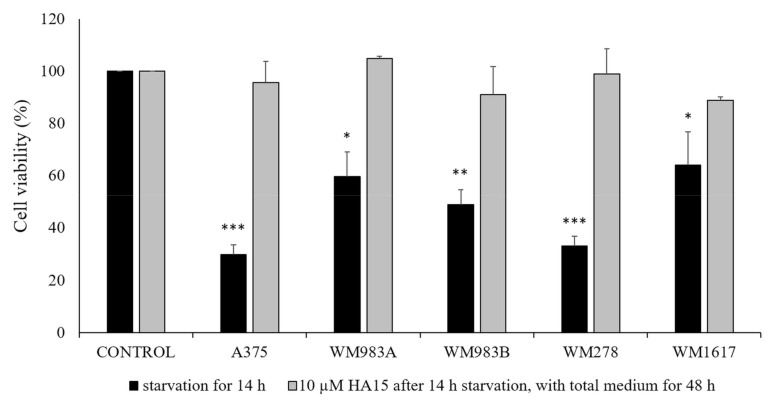
Cell viability of *BRAFV600E*-mutant melanoma cell lines (A375, WM983A, WM983B, WM278, WM1617) treated with 10 µM of HA15 for 48 h. Before drug treatment, the cells were starved for 14 h (black columns); the medium was then replaced with complete medium containing 10 µM of HA15, and the cell viability was measured after 48 h (grey columns) using the WST-1 assay. Data represent the mean viability of three independent experiments ± SD. Significant differences (* *p* ≤ 0.05; ** *p* ≤ 0.01; *** *p* ≤ 0.001) are indicated by asterisks.

During the second experimental arrangement, similarly Cerezo et al. cell lines were starved for 14 h before drug treatment. This condition resulted in a significant decrease in cell viability in all melanoma cell lines (Figure 4, black columns). However, if we replaced the medium with complete medium containing 10 µM of HA15, we observed that the cells recovered completely after 48 h (Figure 4, grey columns), clearly showing that only the starvation condition influenced viability and that the drug had no effect. We observed that the morphology of the cells also changed (Supplementary Figure S1).

**Figure 5:**
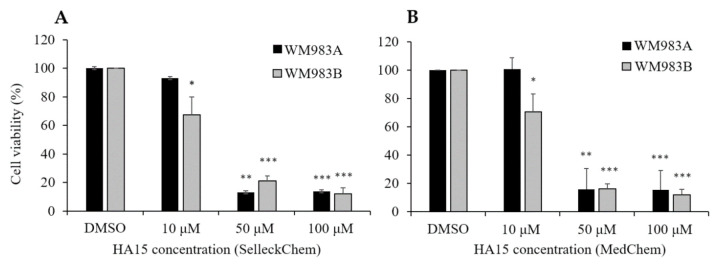
Viability of the WM983A and WM983B cell lines after treatment at different concentrations of drug (10, 50, 100 µM of HA15). HA15 was obtained from two different companies: Sellect Chemicals (**A**) and MedChemExpress (**B**). Before treatment, cells were starved for 14 h; after starvation, different concentrations of HA15 were added to the cell cultures in complete medium. Data represent the mean viability of three independent experiments ± SD and are expressed as percentages of the control. Asterisks indicate significant differences: * *p* < 0.05; ** *p* < 0.01; *** *p* < 0.001.

To strengthen our observations of HA15, which were opposite to the published results, we ordered compound HA15 from another company and repeated the experiments using two cell lines (WM983A and WM983B) with both compounds (SelleckChem Figure 5A; MedChem, Figure 5B). We observed that the viability of WM983A cells did not decrease significantly after 10 µM of HA15 treatment for 48 h but that WM983B cells were sensitive to both compounds, as we detected before. In summary, we observed the same effect for both compounds from different companies. A dramatic decrease in cell viability was observed only at 50 µM and 100 µM of HA15 independently from where the HA15 compound originated (Figure 5A,B).

### 3.3. Effect of HA15 Treatment on Apoptosis Induction in the WM983A Melanoma Cell Line

**Figure 6:**
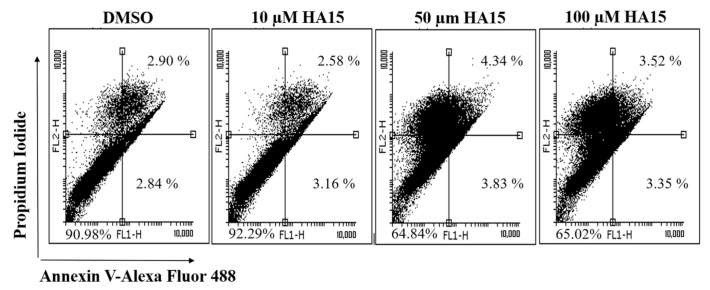
Scatter plots of WM983A melanoma cells after HA15 treatment using the Annexin V FITC/propidium iodide assay. First, cells were starved for 14 h, and the medium was replaced with complete medium containing 10, 50, and 100 µM HA15. The cells were then incubated for 48 h followed by staining with Annexin V-FITC/propidium iodide. The Annexin V-FITC-positive/PI-negative cells located in the lower right quadrant of the histogram represent early apoptotic cells; the Annexin V-FITC-positive/PI-positive cells in the upper right quadrant represent late apoptotic cells.

An Annexin V-FITC apoptosis kit was used to distinguish and quantitatively determine the percentage of apoptotic, viable, dead, and necrotic cells after HA15 treatment. WM983A melanoma cells were starved for 14 h and then treated with various concentrations of HA15 (10, 50, and 100 µM) for 48 h. DMSO (the HA15 solvent) was used as a control. Flow cytometric data are summarized in Figure 6. Based on the flow cytometric data, HA15 treatment did not influence either the cell viability or the rate of apoptosis at 10 µM compared to the DMSO-treated control cells. These data show that HA15 did not induce apoptosis at a concentration of 10 µM (DMSO treatment: early and late apoptotic cells: 5.74%; 10 µM HA15 treatment: 5.74%) and did not affect viability (DMSO: 90.98%; 10 µM HA15: 92.29%). At the same time, higher concentrations of the drug (50 µM and 100 µM) clearly decreased the viability (~65%) (Figure 6).

### 3.4. Effect of Long-Term Starvation on A375 Melanoma Cell Line Viability

**Figure 7:**
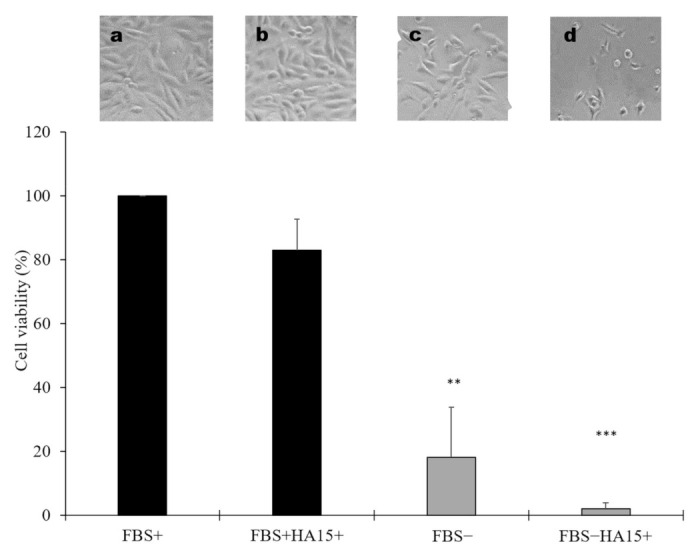
Viability of A375 cells under different cell culture conditions. Before drug treatment, cells were cultured without FBS for 14 h. Then, the cells were treated with 10 µM of HA15 with (FBS+HA15+) or without FBS (FBS−HA15+) for 48 h and the cell viability was determined. Images above the columns show the morphological changes in A375 melanoma cells under different cell culture conditions: (**a**) FBS+: cells grown in complete medium; (**b**) FBS+HA15+ complete medium and 10 µM of HA15; (**c**) FBS– cells grown without FBS; (**d**) FBS−HA15+-starved cells treated with 10 µM of HA15. Data are presented as the mean ± SD of three independent experiments. Significant differences are indicated by asterisks (** *p* < 0.01; *** *p* < 0.001).

To investigate the effect of long-term starvation on cell viability (similar to the published cell culture conditions), A375 cells were starved for 14 h and then cultured with and without FBS for more 48 h as well as with 10 µM of HA15. Although the morphology of the cells was not influenced by the drug treatment (Figure 7a,b) under normal culture conditions, after 62 (14 h + 48 h starvation; grey column) hours of starvation the cell viability decreased significantly (Figure 7c). Indeed, adding 10 µM of HA15 to starved A375 cells resulted in a tremendous decrease in their viability, similar to the published data (Cerezo et al.), and most of the cells were dead (Figure 7d), indicating that long-term starvation and HA15 have synergistic effects on cell viability (Figure 7).

### 3.5. Effect of Serum Withdrawal and HA15 Treatment on Stress Marker Expression in the A375 Melanoma Cell Line

**Figure 8:**
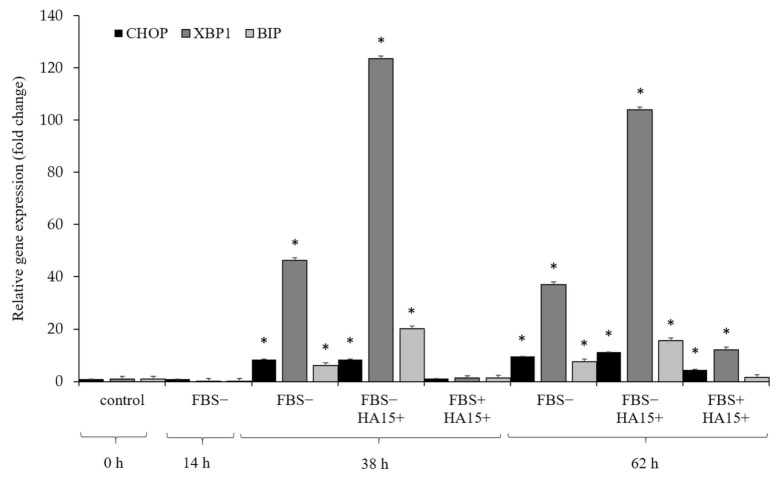
Relative gene expression patterns of stress markers in A375 melanoma cells under different culture conditions. Cells were starved for 14 h and then incubated as follows: without FBS (FBS−), without FBS or with 10 µM of HA15 (FBS−HA15+), or with FBS and with 10 µM of HA15 (FBS+HA15+) for 24 h (14 + 24 = 38 h) and 48 h (14 + 48 = 62 h). Significant differences are indicated by asterisks (* *p* ≤ 0.05).

The level of ER stress was investigated under different experimental conditions at different time points in the A375 cell line. The gene expression of three main stress markers (CHOP, XBP1, and BIP) was determined by qRT-PCR. First, the cells were starved for 14 h (without FBS) and then treated (i) without FBS, (ii) without FBS + 10 µM HA15, or (iii) with FBS + 10 µM of HA15 for 24 and 48 h (similar to the published data). The cells were harvested and the gene expression of the three stress markers was determined (Figure 8).

Based on the expression levels of the three stress markers, serum withdrawal did not affect the relative expression after 14 h. However, after 38 h the expression of the markers increased in the starved cells (with or without HA15 treatment); in contrast to the cells grown under normal culture conditions with HA15, only a slight increase was observed at the end of the experiment (FBS+HA15+: 62 h incubation). On the other hand, high expression levels were observed for the XBP1 gene in starved cells (almost 50× fold change: FBS−), and the expression increased significantly after 38 h in the presence of 10 µM of HA15 (fold change ≥ 120×: FBS−HA15+).

### 3.6. Effect of Serum Withdrawal and HA15 Treatment on the Gene Expression of Autophagy Markers in the A375 Melanoma Cell Line

**Figure 9:**
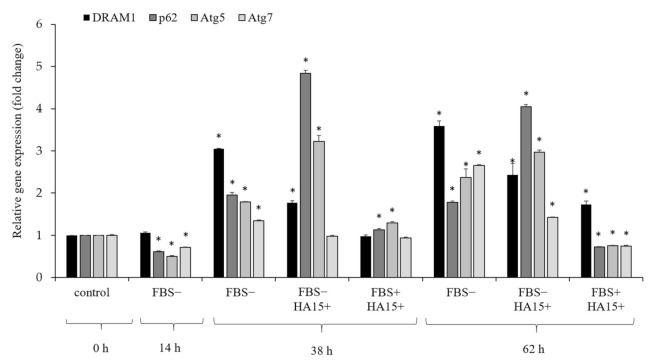
Relative gene expression patterns of autophagy markers in A375 cells. Cells were starved for 14 h and then incubated without FBS (FBS−), without FBS and with 10 µM of HA15 (FBS−HA15+), or with FBS and with 10 µM of HA15 (FBS+HA15+) for an additional 48 h. Gene expression was measured at the indicated time points. Significant differences are indicated by asterisks (* *p* ≤ 0.05).

The induction of autophagy by HA15 was measured using four autophagy-related markers: *DRAM1*, *P62*, *ATG5*, and *ATG7* [19]. We found that at 14 h, pre-starvation (FBS−) did not induce significant expression changes in these genes (Figure 9). After 38 h of starvation, the expression level of all four genes increased (FBS−, 38 h). When incubating the starved cells with 10 µM of HA15 (FBS− and +10 µM HA15), the expression of two genes (P62 and ATG5) further increased, though notable changes in the autophagy marker expression were not observed in cells grown in complete medium, even after 48 h of drug treatment. Overall, the expression patterns of autophagy marker genes were similar in starved cells after 62 h of incubation. In contrast, only the *DRAM1* gene expression was elevated in cells growing in complete medium (FBS+HA15+) after 62 h of incubation.

### 3.7. Development and Characterization of HA15-Resistant Melanoma Cell Lines

**Figure 10:**
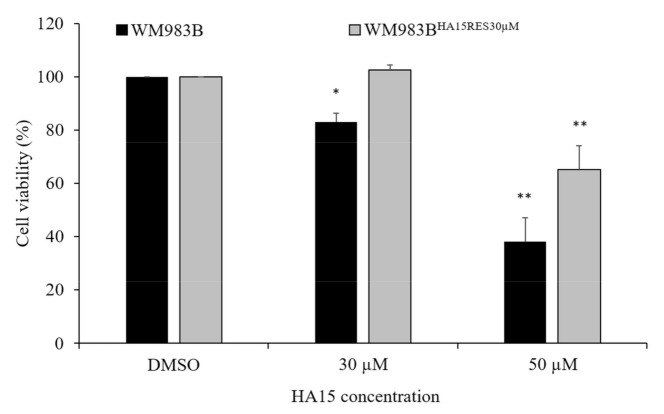
Viability of WM983B and WM983B^HA15RES30µM^ melanoma cell lines. WM983B (black columns) and WM983B^HA15RES30µM^ (grey columns) cell lines were treated with DMSO and 30 µM and 50 µM HA15 under normal culture conditions. Cell viability was measured after 48 h of drug treatment. Data are presented as the mean ± SD of three independent experiments. Significant differences are indicated by asterisks (* *p* ≤ 0.05; ** *p* ≤ 0.01).

To develop drug-resistant cells, we selected the most HA15-sensitive cell line (WM983B) and treated it continuously with 20 µM and 30 µM HA15 under normal culture conditions. After ~10 weeks, two resistant cell lines were produced: one resistant to 20 µM of HA15 (WM983B^HA15RES20µM^) and another was resistant to 30 µM of HA15 (WM983B^HA15RES30µM^) treatment. The viability of the resistant cells (WM983B^HA15RES30µM^) was not affected by continuous HA15 treatment (30 µM of HA15), except when the concentration was increased to 50 µM. Conversely, the parental WM983B cell line showed a significant decrease in viability at both concentrations (Figure 10).

**Figure 11:**
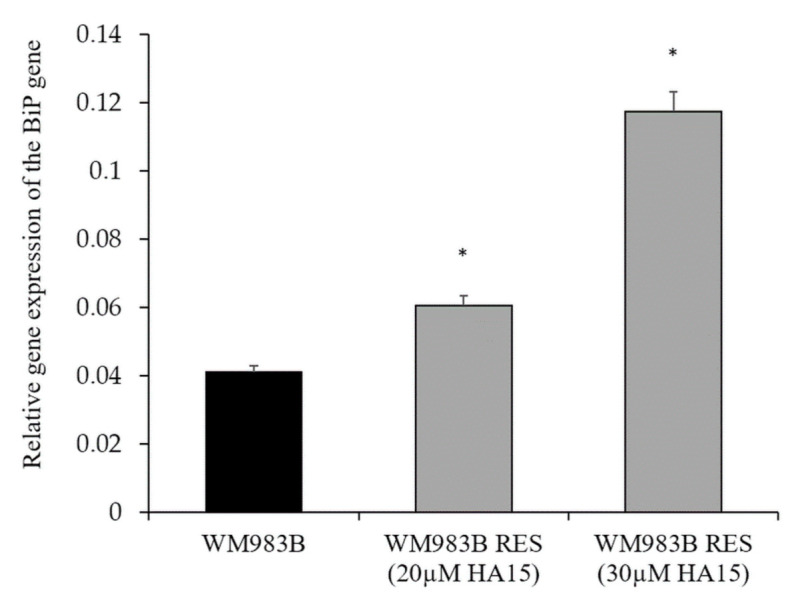
*BiP* gene expression of the WM983B^HA15^-resistant cell line. Two HA15-resistant cell lines were developed by long-term HA15 treatment. The resistant cell lines (grey columns) showed increased *BiP* gene expression compared to the parental cells (black column). Data are presented as the mean ± SD of three independent experiments. Asterisks indicate significant differences (*p* ≤ 0.05).

As HA15 targets the HSPA5/BiP protein (master regulator of the UPR), we determined the expression level of the *BiP* gene in the parental WM983B and WM983B^HA15RES20µM^- and WM983B^HA15RES30µM^-resistant cell lines. Both resistant cell lines showed a significantly increased *BiP* gene expression compared to the parental WM983B cells (Figure 11).

**Figure 12:**
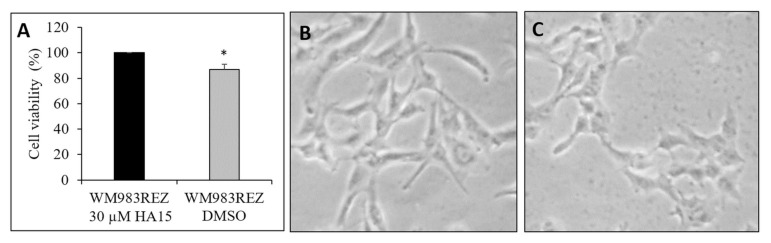
The effect of HA15 withdrawal on the WM983B^HA15RES^-resistant cell line. (**A**) Viability of the cells compared to cells grown in the presence of HA15 (black column) and after the withdrawal of the drug (grey column). The resistant cell lines were cultured in the presence of 30 µM of HA15 or in the presence of the same volume of the solvent (DMSO). After 72 h of drug withdrawal, the WM983B^HA15RES^ cells showed decreased cell proliferation. (**B**) Photomicrographs of the WM983B^HA15RES^ cell line during continuous drug treatment and (**C**) after 72 h of drug withdrawal (100× magnification.). Significant differences are indicated by asterisks (* *p* ≤ 0.05).

We also investigated how the resistant cells (WM983B^HA15RES^) respond to the lack of HA15. During this experiment, we replaced the drug with DMSO and found that the cell viability decreased significantly and that the morphology of the cells changed (Figure 12). The morphological changes of the resistant cells indicates that the cells developed a drug-addiction phenotype, similar to BRAF inhibitor-resistant cell lines (Figure 12B,C) [18].

### 3.8. Effect of Starvation on Gene Expression in the WM983B Melanoma Cell Line Using RNA-Seq Analysis

In the original experiment during the characterization of the anti-cancer effect of HA15, Cerezo et al. used unusual experimental conditions—e.g., cells were starved for 14 h before drug treatment [7]. Therefore, we aimed to determine the gene expression signature of the starved cells and identify differentially expressed genes of cells growing under normal and starvation culture conditions. Based on an RNA-Seq analysis, we detected 6531 upregulated and 4890 downregulated transcripts in WM983B cells growing under starvation conditions. The highest expression difference was observed for the RP11-134F2.8 gene (217-fold change), which encodes a novel DnaJ_C domain-containing protein. The other highly expressed gene was *RP11-618G20.1* (64-fold change), which encodes a long non-coding RNA associated with angiogenesis. Two starvation-related genes, *SLCO4C1* (32-fold change) and *PIK3IP1* (14-fold change), were also highly expressed in the starved WM983B cell population. Based on the molecular functional characterization of the upregulated genes (at least 2-fold changes of 1807 genes), the genes were enriched in calcium ion binding, ion channel activity, and ion transmembrane transporter activity (Supplementary Table S2 and Supplementary Figure S2). Pathways significantly associated with candidate genes in the WM983B melanoma cell line after 14 h of FBS starvation are associated with plasma membrane structures and pathways involved in cholesterol and steroid biosynthesis, calcium signaling pathways, and the activation of gene expression by SREBF pathways (ToppFun GO), as listed in Supplementary Table S3.

### 3.9. Identification of Differentially Expressed Genes in HA15-Resistant Melanoma Cell Lines Using RNA-Seq Analysis

We are the first to successfully establish an HA15-resistant melanoma cell line (WM983B^HA15RES^), which was not particularly difficult because the cell lines were not very sensitive to 10 µM of HA15 treatment under normal culture conditions. However, when the drug concentration was increased to 30 µM, we initially observed decreased cell proliferation; however, after a few weeks the cells were able to cope with the increased amount of HA15 and became a stable resistant cell line. To define gene expression alterations associated with the HA15-resistant phenotype, we performed an RNA-Seq analysis on the newly developed resistant melanoma cell line and compared the gene expression signature to that of the original WM983 cell line.

GO analysis revealed that 2964 of the upregulated genes in the resistant cells are mainly involved in DNA binding, transcription factor activity, and ion channel/transporter activities (Supplementary Table S4); the 2802 downregulated genes are related to the extracellular matrix, integrin, collagen, microtubule, DNA, DNA replication origin binding, and DNA helicase activity and are involved in the cell cycle, mitotic prometaphase, resolution of sister chromatid cohesion, and DNA strand elongation pathways (Supplementary Table S5). We summarize the top 50 overexpressed genes in the WM983B^HA15RES^ melanoma cell line in Supplementary Table S6. The highest expression was detected for the protein-coding gene *PAPPA2* (*Pappalysin 2*), and several genes on the list are specifically linked to drug resistance, such as *ABCC9* and *IL13RA2*.

Using a gene set enrichment analysis (GSEA) depicting the hallmark gene set, we found that the INTERFERON_ALPHA_RESPONSE, HYPOXIA, GLYCOLYSIS, KRAS_SIGNALING_UP, TNFA_SIGNALING_VIA_NFKB, and P53_PATHWAY gene sets correlated positively with the HA15-resistant phenotype (Supplementary Figure S3).

## 4. Discussion

The discovery of mutations of the *BRAF* gene in different types of human tumors has given a huge boost to the development of targeted therapies [20]. The highest frequency of *BRAF* mutations is detected in malignant melanoma, which constitutes a therapeutic target for patients with advanced melanoma [21]. Since the discovery of the *BRAFV600E* mutation, the number of new drugs has expanded dramatically, including effective inhibitors of the MAPK pathway and antibodies targeting immune checkpoint inhibitor molecules, including cytotoxic T-lymphocyte-associated antigen 4 (CTLA-4), programmed cell death (PD)-1, and PD-ligand1 (PD-L1) [4]. These improvements in melanoma treatment have greatly increased the prognosis of patients with advanced melanoma; unfortunately, resistance to most of these treatments limits the number of patients with long-lasting responses. A large number of investigations have focused on identifying the molecular background of resistance, but, regrettably, the leading mechanisms of resistance remain unclear [22]. Therefore, testing all promising treatment options is crucial. Recently, Cerezo et al. described and tested a series of molecules (thiazole benzenesulfonamides), among which the drug HA15 displayed strong anti-cancerous activity in different tumors, including malignant melanoma. The drug was effective in melanoma cells regardless of whether the cells were sensitive or resistant to BRAF inhibitors, whereas no toxicity was observed in normal cells. HA15-induced cancer cell death is mediated by the concomitant induction of autophagy and apoptosis, both of which are the consequence of a rapid initiation of ER stress [7]. Although the anti-cancerous effects of HA15 were well detailed by Cerezo et al., we noticed that the drug treatment conditions for the melanoma cell lines were not optimal [7]. To investigate the effect and to define the molecular target of HA15, during each experiment Cerezo et al. kept the cells under starvation conditions (cell culture without serum) for 14 h before drug stimulation [7]. Because this is not truly a physiological condition, our aim was to investigate the effect of HA15 under normal (complete medium) and starvation culture conditions at different drug concentrations. In addition, we determined the expression of stress (*CHOP*, *XBP1*, and *BIP*) and autophagy (*DRAM1*, *P62*, *ATG5*, and *ATG7*) marker genes; furthermore, we successfully developed an HA15-resistant melanoma cell line. We found that under normal culture conditions, 10 µM of HA15 (this concentration had a deleterious effect on melanoma cells, as described by Cerezo et al.) had only a moderate effect on two of the 10 cell lines, including the A375 melanoma cell line used by Cerezo et al. [7]. Eight of our cell lines (including four BRAF inhibitor-resistant lines) were not sensitive to 10 µM of HA15 treatment at all. In contrast, if cells were cultured without FBS their viability decreased significantly after 14 h of starvation. Under this condition, the cell lines responded differently to HA15: some detached from the surface, and others had rounded shapes, whereas the morphology of other cell lines was unaffected. In fact, we observed the cancer cell killing effect on melanoma cells referred by Cerezo et al. only if we used starvation conditions during the whole experiment [7]. Serum starvation is widely used for mammalian cell synchronization because serum deprivation arrests cells in the G0/G1 phase [23,24,25,26], but after ~24 h (depending on cell lines) the morphology of cells starts to change, as described above. It is well known that starvation results in deleterious effects on cell viability and massive DNA fragmentation [25,27,28,29,30] as a hallmark of apoptosis [31,32]. As FBS is essential for cellular growth and normal metabolism [33], a lack of serum stops cells from proliferating, and their viability decreases due to mitochondrial dysfunction and cytochrome C release, resulting in apoptosis [34,35,36]. How this circumstance affects cell viability, proliferation, and morphology can vary due to the methods used for FBS deprivation and for measuring cell viability [37].

Autophagy is another mechanism that has evolved to provide amino acids and fatty acids during starvation to maintain cell homeostasis; it is compatible with survival and helps cells to cope with stress. Therefore, serum starvation is an easy way to induce autophagy. Many publications have reported that 12 h of starvation is sufficient to increase the number of autophagic vacuoles in cells [38,39,40,41,42]. FBS deprivation causes cell stress in many ways and is the most commonly used method for inducing cell stress [43,44]. For example, it has been reported that HA15 treatment induces ER stress and leads to cancer cell death via concomitant autophagic and apoptotic mechanisms [7]. Nevertheless, the effectiveness of HA15 was determined after 14 h of serum starvation, which would obviously alter the behavior of cells and induce autophagy and apoptotic mechanisms; hence, it is not an ideal experimental background to prove that these mechanisms are induced solely by HA15. To determine the effect of 14 h of starvation on gene expression, we performed RNA-Seq experiments and compared the gene expression patterns in WM983B cells growing in normal growth medium and after 14 h of FBS starvation. The highest difference in expression was detected for the *RP11-134F2.8* gene (217-fold change). This gene encodes a novel DnaJ_C domain-containing protein of the ER that is involved in unfolded protein binding and is an important paralogue of DNAJB11 and a co-chaperone for BiP, which is a master regulator of ER function. BiP is required for proper folding, trafficking, and protein degradation and stimulates BiP ATPase activity [45,46,47]. Another highly expressed gene was *RP11-618G20.1* (64-fold change), which encodes a long non-coding RNA associated with angiogenesis (http://angiogenes.unifrankfurt.de) and has potential clinical implications in ovarian cancer [48]. Two starvation-related genes, *SLCO4C1* (32-fold change) and *PIK3IP1* (14-fold change), were also highly expressed in the starved WM983B cell population. The overexpression of the *SLCO4C1* gene has recently been described in endometrial cancer tissue, and it was shown that the downregulation of this gene can promote apoptosis in endometrial cancer cell lines [49]. Furthermore, the overexpression of *PIK3IP1* suppresses the activity of the PI3K/AKT/mTOR pathway as a critical regulator of autophagy [50,51].

Recent data support the existence of crosstalk between lipid metabolism and autophagy, as lipid metabolism is possibly involved in the formation of membrane structures related to autophagy (e.g., phagophores and autophagosomes) during stress [52]. SREBP is an ER-associated integral membrane protein complex that transcriptionally controls the expression of genes coding for a number of enzymes involved in cholesterol biosynthesis [53]. Similarly, it is well documented that Ca^2+^ ions have a clear and complex role in autophagy regulation [54,55] Finally, the active nuclear form of SREBP1 has been found to amplify ER stress and autophagy via the regulation of PERK [56].

In the materials and method section published by Cerezo et al. [7], the duration of starvation can be interpreted in different ways, whereby 14 or 62 h of starvation may have been imposed if the condition applies to the entire duration of the experiment. If we use cell line models in cancer biology, we must simulate in vivo conditions as “close as possible” to the “biological reality” to obtain useful and reliable information. In contrast to the published data under normal cell culture conditions, we were not able to detect the reported selective, harmful cell killing effect of HA15 at 10 µM. Interestingly, the cell viability decreased significantly after starvation, but if the drug was added to complete medium the cells recovered after 62 h of treatment. In summary, we found that at a small dose HA15 had no significant effect on cell viability, but that the viability of cells decreased significantly at high doses, even under normal culture conditions without starvation.

Finally, yet importantly, contrary to the original publication we were able to generate HA15-resistant cells, which Cerezo et al. was not [7]. The failure to develop an HA15-resistant cell line was interpreted as “it is not surprising since HA15 affects a global cellular mechanism (like ER stress), rather than one specific protein, as the BRAF inhibitors do” [7]. Interestingly, the HA15-resistant cell line shows characteristics similar to those of BRAFi-resistant cell lines [18]. First, after the withdrawal of the drug the cells responded with decreased proliferation, probably because they developed not only resistance to HA15 but also drug addiction. Second, one of the resistance mechanisms to BRAF inhibitors is the overexpression of the target *BRAF* gene [18]. Similar to our previous results, we observed an increased expression of the HA15 target gene *BiP* in a dose-dependent manner in HA15-resistant cell lines. It was recently published that the resistance of cell lines to ER stress inducers is associated with the elevation of GRP78/BiP at the mRNA and protein levels in all resistant cells, suggesting that overexpression of the *BiP* gene might be responsible for HA15 resistance [57].

Moreover, our RNA-Seq data highlight many genes that might contribute to the resistant phenotype. The highest expression in the HA15-resistant cell line developed was detected for the *PAPPA2* gene (more than 900-fold change), though the role of this gene in drug resistance is not yet known; however, it is likely to exert its effects along the insulin-like growth factor (IGF) axis. Nevertheless, several of the other genes identified can be specifically linked to drug resistance. For example, the *ABCC9* gene is a member of the ABC transporter MRP subfamily and is involved in multi-drug resistance, including IL13RA2 in resistance to sunitinib [58] and *ZEB1* in resistance to histone deacetylase inhibitors [59]. The overexpressed genes in the HA15-resistant cell line are involved in ion channel/transporter activities (Supplementary Table S3), and these genes appear to be new actors in chemotherapeutic resistance [60,61].

In summary, in contrast to the published data we conclude that 10 µM of HA15 has only a moderate effect on melanoma cell lines, independent of whether the cells are BRAFi-resistant under normal culture conditions. We showed that the drug-induced anti-melanoma effect is due in part to the starvation applied during the experiments and not exclusively linked to the effect of the drug. Furthermore, we were unable to confirm the selectivity of the HA15—i.e., it similarly influences the viability of normal melanocytes and different melanoma cell lines. In contrast with the published data, melanoma cells are able to develop resistance against the “anti-melanoma” HA15 drug. Finally, our opinion is that further studies are urgently needed to clarify the specific effect of HA15 as an anti-melanoma agent.

## Figures and Tables

**Table 1:** Characteristics of human melanoma cell lines.

Cell Line	Origin ^a^	Growth Phase ^b^	Histologic Type ^c^	BRAF Mutation Status ^d^
WM983A ^p1^	primary	VGP	n.d.	V600E
WM983A ^PLX4720RES^	primary	VGP	n.d.	V600E
WM983B ^m1^	metastasis	-	-	V600E
WM983B ^PLX4720RES^	metastasis	-	-	V600E
WM278 ^p2^	primary	VGP	NM	V600E
WM278 ^PLX4720RES^	primary	VGP	NM	V600E
WM1617 ^m2^	metastasis	-	-	V600E
WM1617 ^PLX4720RES^	metastasis	-	-	V600E
A375	primary	-	-	V600E

^a^ tumor type of melanomas from which the cell lines were derived; ^b^ VGP: vertical growth phase; ^c^ NM: nodular melanoma; n.d.: no data; ^d^ V: valine; E: glutamic acid; ^p1, p2^ primary tumor-derived cell line with metastatic pair from the same patient; ^m1, m2^ metastatic pair of primary-derived cell line; ^PLX4720RES^ BRAF inhibitor (PLX4720) resistant cell lines, all cell lines were wild type for the NRAS mutation.

## Data Availability

The RNA-Seq raw data were deposited into the Sequence Read Archive database: (https://www.ncbi.nlm.nih.gov/geo/query/acc.cgi?acc=GSE164261) under accession number GSE164261.

## Supplementary Materials

The following are available online at https://www.mdpi.com/2227-9059/9/2/96/s1: Figure S1: Morphological changes induced by starvation in the WM983A melanoma cell line. (A) WM983A cell line in normal medium and (B) after 14 h of FBS starvation. Figure S2: Upregulated genes in the WM983B melanoma cell line after 14 h of starvation, as shown in a molecular functionally grouped network using the ClueGo (v. 2.3.5) tool kit of Cytoscape (www.cytoscape.org) software (v. 3.5.1). Figure S3: Gene set enrichment analysis (GSEA) of the WM983B^HA15RES^ melanoma cell line using the hallmark gene set. Table S1: Primer sequences used in RT-qPCR experiments. Table S2: Molecular functional characterization of upregulated genes (6531 genes) in the WM983B melanoma cell line after 14 h of FBS starvation. Table S3: Significantly altered pathways in the WM983B melanoma cell line after 14 h of FBS starvation using ToppFun Gene Ontology. Table S4: Groups of upregulated genes by molecular functions in the WM983B^HA15RES^ melanoma cell line using ToppFun Gene Ontology. Table S5: Groups of downregulated genes by molecular functions in the WM983B^HA15RES^ melanoma cell line using ToppFun Gene Ontology. Table S6: Top 50 overexpressed genes in the WM983B^HA15RES^ melanoma cell line.
