# Supplementary figures and images for: Cell Proliferation Is Strongly Associated with the Treatment Conditions of an ER Stress Inducer New Anti-Melanoma Drug in Melanoma Cell Lines
Source: Biomedicines. 2021 Jan 20;9(2):96. doi: 10.3390/biomedicines9020096 (PMC7908983; doi:10.3390/biomedicines9020096)

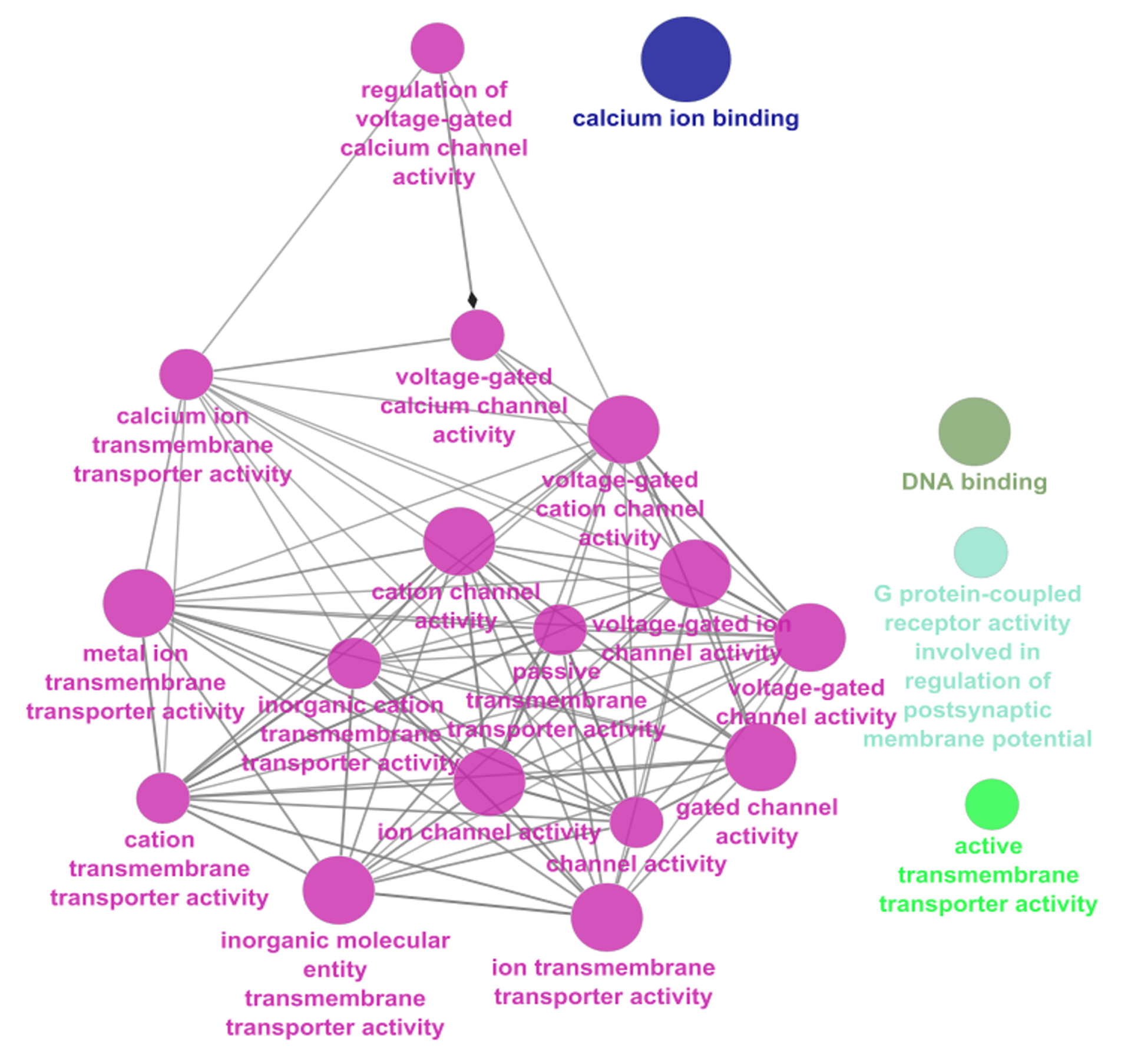

Supplement: Supplementary file 1 [file biomedicines-09-00096-s001.zip › Supplementary Figure_2_Szasz.tif]

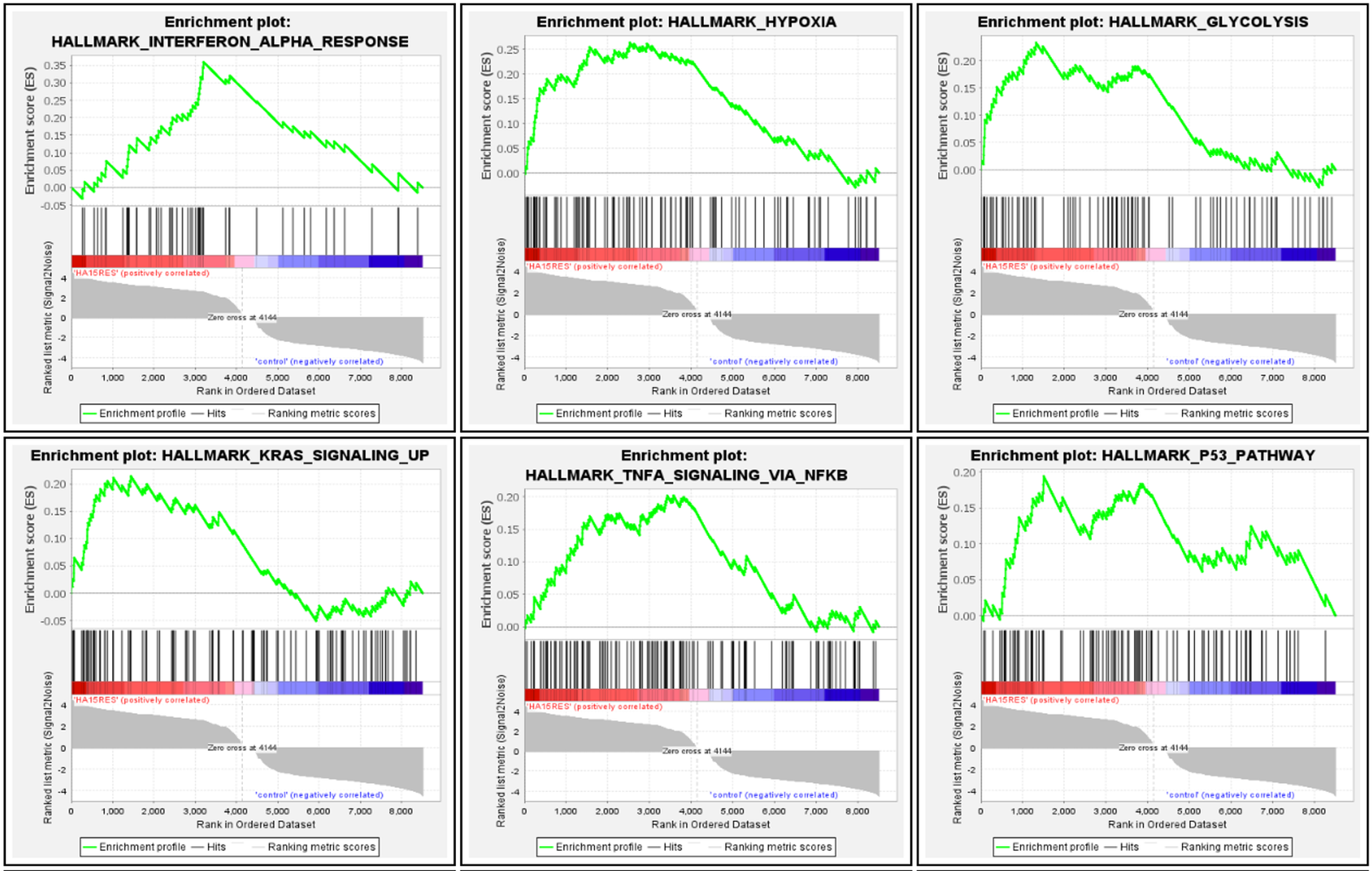

Supplement: Supplementary file 1 [file biomedicines-09-00096-s001.zip › Supplementary Figure_3_Szasz.tif]
